# Assessment of health professional education across five Asian countries—a protocol

**DOI:** 10.1186/s12960-018-0316-6

**Published:** 2018-10-03

**Authors:** Sanjay Zodpey, Pisake Lumbiganon, Tim Evans, Ke Yang, Bui Thi Thu Ha, Himanshu Negandhi, Wanicha Chuenkongkaew, Ahmed Al-Kabir

**Affiliations:** 1 Public Health Foundation of India, Delhi, NCR India; 2 Department of Ob & Gyn, Faculty of Medicine, Khon Kaen University, Khon Kaen, Thailand; 3 World Bank, Washington, DC United States of America; 4 Peking University Health Science Center, Beijing, China; 5 Hanoi School of Public Health, Hanoi, Vietnam; 6 Department of Ophthalmology, Faculty of Medicine, Siriraj Hospital, Mahidol University, Bangkok, Thailand; 7 Research, Training and Management (RTM) International, Dhaka, Bangladesh; 8 Indian Institute of Public Health, Public Health Foundation of India, Plot No 47, Sector 44, Institutional Area, Gurgaon, Haryana 122 002 India

**Keywords:** Health professional education, Asia, Competencies

## Abstract

**Background:**

There is an increasing consensus globally that the education of health professionals is failing to keep pace with scientific, social, and economic changes transforming the healthcare environment. This catalyzed a movement in reforming education of health professionals across Bangladesh, China, India, Thailand, and Vietnam who jointly volunteered to implement and conduct cooperative, comparative, and suitable health professional education assessments with respect to the nation’s socio-economic and cultural status, as well as domestic health service system.

**Methods:**

The 5C network undertook a multi-country health professional educational study to provide its countries with evidence for HRH policymaking. Its scope was limited to the assessment of medical, nursing, and public health education at three levels within each country: national, institutional, and graduate level (including about to graduate students and alumni).

**Results:**

This paper describes the general issues related to health professional education and the protocols used in a five-country assessment of medical, nursing, and public health education. A common protocol for the situation analysis survey was developed that included tools to undertake a national and institutional assessment, and graduate surveys among about-to-graduate and graduates for medical, nursing, and public health professions. Data collection was conducted through a mixture of literature reviews and qualitative research.

**Conclusions:**

The national assessment would serve as a resource for countries to plan HRH-related future actions.

## Background

The Flexner report sparked groundbreaking reforms in health professional education leading the integration of modern science into curricula of university-based medical schools at the turn of last century. The reforms in health professional education are credited with equipping health professionals with knowledge that contributed to the doubling of life span during the twentieth century [1]. Health professionals worldwide have to adequately address health problems of the public through the provision of quality health services, which should be universally accessible and ultimately lead to improvement in the lives of people globally. It is imperative that healthcare advances and lessons for improvement are systematically collated; evidence-based learning is shared across settings, both domestically and internationally, and policymaking are aligned to the contextual needs of populations and countries. The Future of Nursing report and the Lancet Commission report on Education of Health Professionals have brought the issue of health professional education in the limelight. Healthy populations and well-performing national health systems are fundamental for equitable, inclusive, and sustainable development [2].

Health professional education belongs to the supply side of health systems but is in a state of crisis. Health professional education is poorly adapted to address health system challenges, largely, because of outdated, static, and fragmented curricula that produce graduates with insufficient knowledge, skills, and competency responsive to the present and future population and communities’ health needs [3]. There is also increasing consensus globally that the education of health professionals is failing to keep pace with scientific, social, and economic changes transforming the healthcare environment. Since the initiation of the joint work on health professional education by WHO and The US President’s Emergency Plan for AIDS Relief (PEPFAR) in 2009, and discussion on the Agenda for Global Action on Scaling up Health Worker Education and Training by the second global forum on Human Resources for Health at the Prince Mahidol Award Conference 2011 [4], a new movement to tackle current inadequacies of health professional education gathered pace across a range of different stakeholders [5]. The “Commission on Education of Health Professionals for the 21st Century,” which was established and chaired by Professor Lincoln Chen (President of China Medical Board) and Professor Julio Frenk (Dean of Harvard School of Public Health), launched a report on “Education of health professionals for the 21st century: a global independent commission.” This catalyzed a solid movement in reforming education of health professionals. For example, Bangladesh, China, India, Thailand, and Vietnam jointly volunteered to implement and conduct cooperative, comparative, and suitable health professional education assessments with respect to the nation’s socio-economic and cultural status, as well as domestic health service system.

A regional network on HRH education was initiated by the five countries during a Hanoi consultative meeting in 2011 to promote the exchange of knowledge, information sharing, and learning in health profession education (medical, nurse, or public health professional) reform including internationally collaborative activities. The Vietnam team coordinated the initial activities of the network. The Thailand team coordinated a multi-country situation analysis survey of HRH education. This regional network was named as the Asia-Pacific Network on Health Professional Education Reform (ANHER) with a plan to formulate the promotion of knowledge exchange, information sharing, and learning in health profession education (medicine, nursing, and public health) reform including internationally collaborative activities [6]. This five country regional network (5C network) comprises of Bangladesh, China, India, Thailand, and Vietnam and provides a platform for collaborative activities in the region. The overall goal for this network is “to develop and strengthen the Asia Pacific regional network of health professional training institutes as a platform for collaboration on knowledge synthesis and evidence generation for health professional education reforms movements in response to changing health determinants and health systems development in countries.”

## Methods

The 5C network undertook a multi-country health professional educational study to provide its countries with evidence for HRH policymaking. Representatives from the five member countries met over a series of meetings to define the goal and objectives of the study, discuss appropriate design for the cross-country assessment, and create common study protocols and tools. The scope of the 5C study was limited to the assessment of medical, nursing, and public health education at three levels within each country: national, institutional, and graduate level (including about to graduate students and alumni). All countries planned to undertake the assessment of the three professions (medicine, nursing, and public health) at all the three levels with the exception of Thailand that only participated in the assessment of medicine and nursing professions. The five member countries agreed that the overarching principle of the assessment was to generate evidence to support policymaking that is contextual to the needs of the country, comprehensive in creating a complete picture of the education of health professionals at country level. The health professional education assessment (across institutional and instructional domains) was guided by the framework suggested by the Lancet Commission for transforming education to strengthen health systems in an interdependent world [1].

A common protocol for the situation analysis survey was developed that included tools to undertake a national and institutional assessment, and graduate surveys among about-to-graduate and graduates for medical, nursing, and public health professions. The protocols were kept flexible to permit each of the five countries to customize responses to questions so as to better reflect their country context. The tools for each level of assessment were designed to focus on specific questions such as:

- National level including descriptions of:
  - ○ Country scenario through national basic indicators;
  - ○ National policy for higher education of health professionals;
  - ○ Communication and inter-sectoral coordination among ministries, especially Ministry of Education [MOE] and Ministry of Health [MOH];
  - ○ Assessment of demand and supply for HRH;
  - ○ Institution accreditation;
  - ○ National standards for curriculum;
  - ○ Innovative education training policies and outcomes;
  - ○ Perspectives and viewpoints from senior peers in public/private, urban/rural hospitals on graduate’s clinical competencies, management skills, communication skill, inter-professional skills, and orientation towards working with public systems and ethics through FGDs.
  Data collection was conducted through a mixture of literature reviews and qualitative research. The national assessment would serve as a resource for countries to plan HRH-related future actions.
- Institutional level related to:
  - ○ Institutional governance;
  - ○ Educational services (covering curriculum analysis, evaluation of core competencies of students);
  - ○ Workforce of faculties;
  - ○ Financing;
  - ○ Infrastructure and technology;
  - ○ Information for policymaking; and
  - ○ Quality assurance in education.

Questions were adjusted according to country context, while retaining several core questions for cross-country comparisons. The respondents from schools were assisted by research associates/study team members to complete these questionnaires.

- Graduate survey focused on assessing perception and attitude about educational system, including medical, nursing, and public health students just about to graduate and doctors/nurses/PH professionals in service, i.e., alumni. This graduate assessment covered:
  - ○ Socio-demographic background of the graduates and their parents;
  - ○ Perception/attitudes towards rural, remote, or hardship areas;
  - ○ Job preferences upon graduation and 5 years later;
  - ○ Competency self-assessment;
  - ○ School facilities assessment; and
  - ○ Student financial issues.

Self-administered questionnaire were used for this survey.

The sample size for the cross-sectional study is presented in Table 1, and the proposed sampling structure is included in Table 2.

**Table 1:** Sample size for cross-sectional study

		Sample size				
		India	Bangladesh	Vietnam	China	Thailand
Public health	Institutional assessment	23	14	5	10	NA
	Public health graduate	100	164	125	721	NA
	Public health alumni	100	55	125	208	NA
Nursing	Institutional assessment	80	27	10	16	40
	Nursing graduate	1500	831	400	2218	3349
	Nursing alumni	1500	226	400	452	475
Medicine	Institutional assessment	35	23	8	17	19
	Medical graduate	1500	1 422	225	3045	1238
	Medical alumni	1500	207	225	824	570

*NA* not applicable

**Table 2:** Sampling for the cross-sectional study

	Medicine	Nursing	Public health
Bangladesh	Stratified random sampling by geography	Stratified random sampling by geography	All schools
China	Stratified random sampling and purposeful sampling	Stratified random sampling and purposeful sampling	Stratified random sampling and purposeful sampling
India	Stratified random sampling by geography	Stratified random sampling by geography	Stratified random sampling by geography
Thailand	All schools	Stratified random sampling by ownership	NA
Vietnam	All schools	Stratified random sampling by geography	All schools

The tools were originally developed in English for Bangladesh and India, while China, Thailand, and Vietnam translated the final tools into their local language. A common codebook and a centralized data management system were evolved, and Thailand led the effort for creating a centralized data management plan. Individual countries shared anonymized data, and the secretariat in Thailand did data cleaning. The data analysis plan was developed during a meeting held at Dhaka in 2013. The study was approved by institutional review boards/ethics committees in each of the five countries. All five countries pilot tested the revised tools at least one medical school, one nursing school, and one public health school before conducting the study.

## Results

The crisis in health professional education needs urgent attempts to undertake institutional and instructional reforms that comprehensively address systemic issues plaguing the very structure and functioning of systems tasked with creating competent health professionals. An increased attention is accorded to health professional education after this issue was prominently highlighted at the global level through the Joint Learning Initiative [7], the 2006 World Health Report [8], and the Lancet Commission on Education of Health Professionals [1]. Since health professional education is an integral component of health workforce development, it can also be expected to strongly influence the global movement towards universal health coverage and health equity. The 66th World Health Assembly in May 2013 had also adopted a resolution WHA66.23 on transforming health workforce education in support of universal health coverage. Although transformative learning is vital towards advancing reforms in health professional education and its philosophy is well-understood, the evidence on its actual design, implementation, and collaborative action are still lacking. WHO recognizes that *reforms in education must be informed by community health needs and evaluated with respect to how well they serve these needs* [5] and is addressing the technical dimensions that can bring about a new era for health professional education [5]. It is important for countries to understand their healthcare profile, contextualize current efforts towards health professional education reforms, and document existing successes.

This paper presents the protocols that were used in a five-country assessment of health professional education that encompasses medical, nursing, and public health education. This study is the largest and most comprehensive assessment of health professional education ever undertaken globally and provides an opportunity for countries to meaningfully engage with their policymakers through the creation of evidence to understand their current situation and sound decision-making. Of the five countries participating in this network study, three countries (China, India, and Bangladesh) are in the top 10 most populated countries of the world needing a large health workforce to meet health needs of their population [9]. The global HRH scenario is plagued by inequities with a higher health worker: population ratio in developed countries viz. developing countries which have a much lower health worker: population ratio [8, 10]. There is a health worker migration from developing to developed countries; and within countries from rural to urban areas. At the country level, a critical shortage of health workforce is an immediate health systems issue in the South East Asia region with five (Myanmar, Indonesia, Bhutan, Timor-Leste, and Bangladesh) out of 11 member states facing a critical shortage of health workforce [2]. The regional average is slightly below the benchmark of 22.8 doctors, nurses, and midwives per 10 000 population [2]. The WHO 2010 global policy recommendations on interventions to improve attraction, recruitment, and retention in remote rural areas report either a “low” to “very low” for the quality of evidence for action across interventions in the domains of education, regulation, financial incentives, and professional and personal support [11]. This lack of evidence adds to the inertia of policymakers in making an informed choice even for interventions that are backed by a strong recommendation.

The results of the five-country effort have been published earlier [12, 13] highlighting that medical students’ low positive attitudes towards their school in inspiring them to work in rural area as well as their low confidence in overall competency to work in rural area should strongly alert administrative authorities of medical schools [13] and appropriate strategies including more emphasis on community and competency-based learning should be implemented based on local context [13]. Another paper highlighted that nursing students with rural upbringing and recruitment had more positive attitudes towards rural areas and were more likely to choose working in rural areas after graduation [12].

The supply side of health systems is poorly documented globally. Our study was conducted at three levels: national, institutional, and individual (graduates) for a comprehensive assessment of the education situation across policymaking, implementation, and outcome of current health education system of the member countries. We therefore consider our study to be holistic by attempting to outline the current situation of national, institutional, and graduate perspective for understanding the supply of HRH. There have been only limited attempts within individual countries with a piece-meal descriptive analysis of the supply side of health education systems [14] before our study. Since this work was the first of its kind, the research team had to contend with the creation of valid and standardized tools and methodology for completing this situational assessment. Over multiple consultations, the research team from all five countries arrived upon a methodology that was aligned with the framework as recommended by the Lancet Commission report for education of health professionals.

The study provides a common platform for participating research institutions within these five countries to build their capacities in undertaking a large multi-country health professional education assessment. The systematic assessment of the national scenario, institutional and graduate assessment can help identify the missing pieces of the HRH puzzle.

There are a number of innovations on health professional education interventions, such as recruitment of secondary school students from rural areas for nursing and medical education in Bangladesh, laddering approach of production of nurses [such as 2 years training, post in rural hospitals for a few years, and continued training for years 3 and 4 for a professional nurses, upgrading training of medical assistants for a physician], rural retention strategies such as mandatory government bonding for health professional graduates, additional financial and non-financial incentives for health workers in rural areas, different innovative training such as inter-professional education, exposure of nurse and medical students to rural communities, problem-based learning and continued professional development. An assessment of these interventions, what works and what does not work, and documenting good practices are essential to support scaling up effective interventions in a country. This study provides a platform for an assessment of these innovations by capturing individual country experiences and presenting them to the other countries in the network for cross-learning.

## Discussion

ANHER serves as a regional platform, which facilitates sharing, and exchanges of effective interventions and good practices among health professional education institutes in the region and encourages south-south collaboration among countries sharing common HRH problems. These good practices and interventions can be considered by each country/institution to modify, and apply for small-scale piloting in different settings and scale up as appropriate among countries in the region. The protocol encourages each country to gain ownership of the reform agendas by key stakeholders and ensure the uptake of research findings and involve a joint assessment by researchers from inside and outside the schools. The protocol encourages research in collaboration with ministries, government bodies, accreditation councils, and other important policy stakeholders from study planning to dissemination.

As health professional education is complex, it consists of various stakeholders in each country; therefore, networking among researchers inside and outside the schools and other key stakeholders such as Ministry of Health, Ministry of Education, health professional councils and associations, public and private sector employment sector in a country is vital for success in health professional education reform.

**Table:** 

Unique features of our study: • Large multi-country study • Developing country and LMIC focus • Covers three critical professions (medicine, nursing, public health) • Comprehensive assessment across three levels: country, institution and graduate • Development of new tools in consultative mode • Guided by representatives of national governments	

The dissemination plan/implications for our work include organizing national meetings/conference and engagement of professional councils and establishing government partnerships and conference presentations. Countries planned a series of consultation meetings with different types of stake holders as well as a national level dissemination.

Our work was limited by circumstances that limited the design of a uniform tool that could be used across all five countries for the assessment at each level for individual professions. Herein, we adopted the principle of permitting countries to introduce questions to better reflect their context or include questions that policy makers in their country wanted to address. These tools were additionally used in local language across some countries, adding one additional source of potential bias. We did not directly standardize the trainings for data collectors but expected countries to standardize the trainings for data collectors within their own countries.

## Conclusions

The study provides a platform for participating institutions within five countries to build their capacities in undertaking a large multi-country health professional education assessment. The systematic assessment of the national scenario, institutional, and graduate assessment can help countries to plan HRH-related future actions.

Our protocol is also backed by a strong regional imperative wherein the WHO Regional Committee for South-East Asia passed a resolution SEA/RC65/R7 in September 2012 [8] for strengthening health workforce education and training in the region. The Regional Committee urged member states to review national health workforce policies, strategies, and plans to maximize their contributions to the health of the population and the achievement of universal health coverage; to conduct comprehensive assessments of the current situation of health workforce education and training, based on an agreed regional common protocol, as a foundation for evidence-based policy formulation and implementation; and to develop or strengthen policies for education and training of the health workforce as an integral part of national health and education and training policies.
